# Supplementary material for: Mutations in PCYT2 disrupt etherlipid biosynthesis and cause a complex hereditary spastic paraplegia
Source: Brain. 2019 Oct 22;142(11):3382–97. doi: 10.1093/brain/awz291 (PMC6821184; doi:10.1093/brain/awz291)
Supplement: awz291_Supplementary_Data [file awz291_supplementary_data.zip › awz291-Suppl_data/Supplementary_Data4.zip!/Supplementary information 3 - fibroblast lipidomics.zip!/Fibroblast lipidomics/Patient1 vs Patient2/Statistical plots and data/TG[O] species/Boxplots all.pdf]

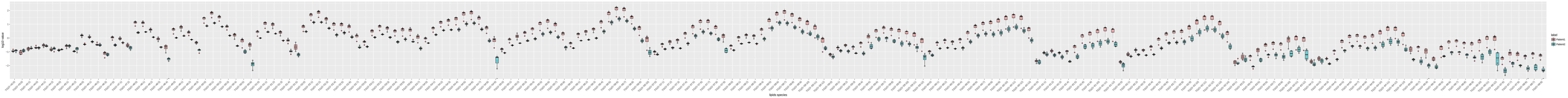

log10 value
2
1
0
−1
−2
TG(O−42:0)
TG(O−43:0)
TG(O−43:1)
TG(O−44:0)
TG(O−44:1)
TG(O−44:2)
TG(O−45:0)
lipids species
label
Patient1
Patient2
